# What’s in the box? Exploring UK players’ experiences of loot boxes in games; the conceptualisation and parallels with gambling

**DOI:** 10.1371/journal.pone.0263567

**Published:** 2022-02-09

**Authors:** Sarah E. Hodge, Max Vykoukal, John McAlaney, Reece D. Bush-Evans, Ruijie Wang, Raian Ali

**Affiliations:** 1 Department of Psychology, Bournemouth University, Poole, United Kingdom; 2 College of Science and Engineering, Hamad Bin Khalifa University, Doha, Qatar; University of Electronic Science and Technology of China, CHINA

## Abstract

Loot boxes are a popular mechanic within many video games, but it remains unclear if some forms of loot boxes can be seen of as gambling. However, the perspectives of players are often neglected, such as whether they see them as ‘fair’ game elements and how closely they feel this aligns with gambling. In this paper, we synthesise a conceptualisation for loot boxes through players’ actual experience and explore if there are any parallels with gambling. Twenty-one participants who played video games took part in the research through either an interview or online survey. Thematic analysis suggested that six themes were core to exploring loot boxes: Random Chance Effects, Attitudes Towards Content, Implementation, Parallels with Gambling, Game Design, and The Player. The results suggested both indirect and direct parallels with gambling from the players experiences. Implications of game design and classifying loot boxes as gambling are discussed in relation to game design and risk factors of gambling and purchasing behaviour.

## Introduction

With over 3 billion active players worldwide and a market exceeding $150 billion in 2021 alone, gaming is a popular activity [1]. Along with its expanding popularity, concerns have been expressed about excessive play, game mechanics and safeguarding individuals from potential harms [2–4]. Such concerns have led to the inclusion of age ratings on games [2], updated government frameworks (e.g. [5]) and industry recommendations for safeguarding players [6]. Yet, research and policy regarding an increasingly popular consumable virtual item in games, such as loot boxes require much more attention. In video games, a loot box is a consumable virtual box which can be redeemed to receive or ‘win’ randomised in-game items from a selection of options [7]. These items may be aesthetic options for a player’s avatar or support mechanics to help enhance progress within the game [8]. Even before the terminology of loot boxes was used, games would reward players in a variety of ways [9]. Hence, loot boxes have evolved from: how games reward players, Massively Multiplayer Online Role-Playing Games (MMORPG) and the monetisation of free-to-play mobile games [9]. They are seen by developers and publishers of video games as economic opportunities (i.e. in-game purchases or microtransactions) and ways to keep players interested within games [10–13]. Indeed, in 2020 loot boxes were prevalent in over half the top grossing games available on Android and iOS devices [14].

For many games and within video game history, an inherent part of the gameplay is acquiring in-game items via treasure /loot through chest/box elements [15]. For example, the majority of The Legend of Zelda [16] series of games contain treasure chests containing hidden objects/items, which require in-game money/currency or ‘keys’ to unlock them. Although the items revealed may appear random from the player’s perspective, they are often predetermined by game developers. This has led to questions around the lack of transparency from gaming companies in the algorithms used in loot boxes (which are often not disclosed) as well as in their strategies to use them to create competitive advantage [17]. Then, from a player’s perspective, they may feel lured and at times pressured to buy them [18–20]. Given that loot boxes have been described as virtual treasure boxes [21], it is possible that they have evolved from traditional game features (e.g. in Role Playing Games (RPGs)) such as those within The Elder Scrolls series [22]. An example of the randomness element seen from acquiring in-game items (i.e ‘loot’) is the Diablo series [23], whereby the game includes a loot drop feature in which in-game items randomly appear to the player [9]. However, there are some differences from this traditional game feature as loot boxes tend to emerge in online games and require real money to access them. Such ‘pay-to-win’ gameplay is often likened to compulsion loops that are aimed to keep players invested in a game and are frequently linked to gambling addiction [24]. Within the literature, the use of the term loot boxes has been criticised for their lack of acknowledgment of the game mechanics and some argue that Random Reward Mechanics (RRMs) should be adopted instead as these underlie the random nature of rewards: those that can’t be purchased, those that can only be sold, those that can only be purchased and those that can be both purchased and sold [9]. Particularly the last one, ‘those that can be both purchased and sold’ the authors argue relates and is equivalent to gambling [9].

### Loot boxes: Features and design

**Fig 1:**
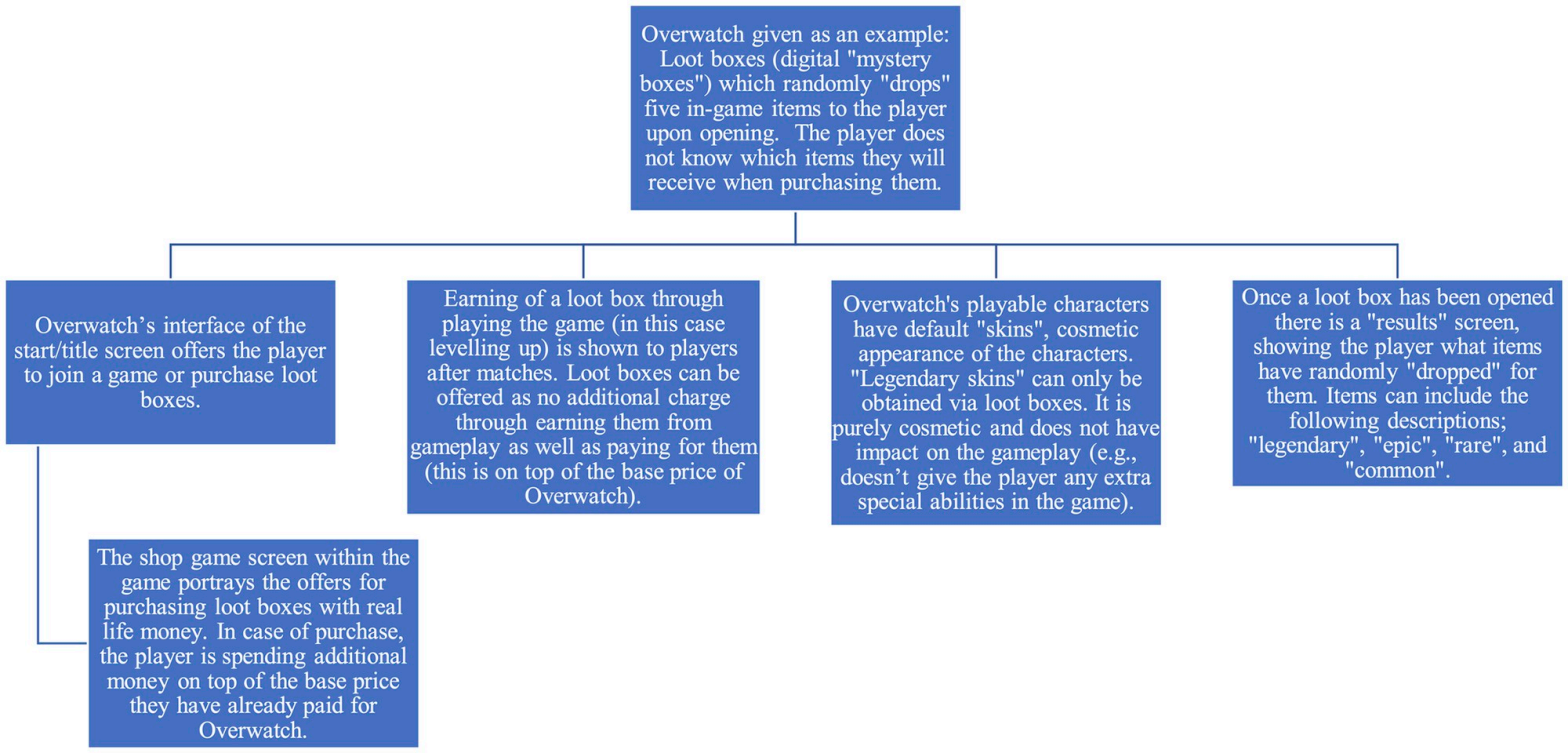
The structure of Overwatch loot boxes [25].

One popular video game that features loot boxes is Overwatch [25]. Overwatch is played on PC, PlayStation 4, Xbox One, and Nintendo Switch platforms [8]. See Fig 1 for an outline and description of loot boxes in this game.

It is important to consider that loot boxes do not necessarily appear in a ‘box’ format. For example, Hearthstone [26] is a free to play online strategy and collectable card game where players collect cards to support winning and progression in the game. A player progresses in Hearthstone [26] by building strong card decks with which they can beat opponents. The more cards a player has, the more likely they are to be able to build a strong deck (i.e., there is the more strategic choice to adapt to the current game). Hearthstone [26] gives the player the options to buy a set of five random cards. Before the player buys the cards they are informed by the game that one of the five cards will be rare, suggesting some predictability to the random nature of the purchase and loot boxes. This game is dynamic and the cards frequently change in usefulness. Having a high number of cards supports winning and also prevents boredom stemming from playing continuously with the same cards.

### In-game gambling

There are games which mimic forms of gambling including arcade and casino games, one example is Coin Pusher [27] which is a virtual replication of the arcade coin pushing machines. In-game gambling can consist of a variety of games including Poker, Roulette, Blackjack, and Slot Machines. For example, in their extension of Grand Theft Auto (GTA) [28], Rockstar Games offers the player the ability to experience a casino and resort within the game as an avatar. Such game play leans towards scholarly discussion relating to in-game gambling that can be experienced in the game itself and real-life gambling. The boundaries between gambling within the game and real-world gambling are further blurred by players being able to use real money to purchase in-game currency that can be used for in-game gambling. In addition, although GTA [28] has an adult rating, it has been found to be popular with adolescents [29].

With regards to gambling mechanisms in these games it is important to highlight that once the player has purchased the product, they cannot redeem real-life money for it, and it remains an in-game purchase. However, in other games, products including skins (cosmetic items for a player’s avatar/game character (e.g. [30])) and in-game items can be sold and/or redeemed for in-game money. Furthermore, although in-game items normally cannot be redeemed for real-life money, this may not eliminate second-hand markets for these items (e.g. selling skins from Overwatch [26]), especially from the reports of the players perceiving these items as valuable/desirable even items that do not require real world money to purchase can still have real world value [31, 32]. Additionally, even though game companies may not permit cashing out, the perceived desirability/value of the items obtained could facilitate cashing out behaviours and even pose legal and criminal risks to the player, [33]. Electronic Arts (EA) tried to combat cashing out in their FIFA games [33] from the term and conditions of play, but this did not deter players from this behaviour (e.g. using third party websites) [34]. In contrast to gambling, where cashing out for real money can take place. The mechanisms of loot boxes can be applied to real-life forms of traditional gambling, such as raffles and lotteries where people buy tickets for the opportunity to win money/prizes. The UK Gambling Commission [35] has stated that raffles and lotteries are forms of gambling and recommended restriction on the monetary value of prizes and expenses that can be claimed as well as a license being required. These existing traditional gambling regulations highlight the support for players (e.g. to reduce harm and provide care) and therefore, regulation needs to be considered where there may be overlaps with gambling or similar mechanisms/features, such as loot boxes.

Gambling on electronic gaming machines (e,g. slot machines) has been suggested as one of the risk factors for developing problem gambling; as well as previous gambling behaviour on table games, races, sports or lotteries for males; and scratch tickets and bingo for females [36]. Additionally, research has found a relationship between purchasing loot boxes and other in-game gambling type behaviours (e.g. Esports betting, social casino games, transactions with real money, and token wagering) were connected to problematic gambling and gaming behaviours [37]. Unlike traditional forms of gambling in sports and some casino games, for online/virtual games, the chances of winning is known and programmed by the developer through algorithms into the game, but not necessarily known to the player. This raises questions around transparency of the chances/odds of winning items from loot boxes, particularly the degree of fairness. For example, developers could make a game deliberately boring and nudge players towards loot boxes as in-game accelerator mechanisms. This has led to the observation that loot boxes are predatory and aim to manipulate the player [38] or seen as a ‘sludge’, a dark form of nudging [17]. Hence, the People’s Republic of China have legislated that the probabilities/chances of obtaining items from loot boxes is disclosed, in which Xiao et al. suggest should be a standard requirement of loot boxes [17], with ethical design considerations [39]. More research is needed to explore the role of long-term effects of the exposure to loot boxes and in-game gambling. King and Delfabbro [11] suggested that young people could be at higher risk of developing problem gambling later in life from simulated gambling, although a causal and transitional link has not been previously reported or suggested for young people in the UK [40].

### Prevalence of gaming and gambling in young people

In their 2019 report on gaming and gambling in young people (aged 16–24), the UK charity Young Gamers and Gamblers Education Trust (YGAM) identified that 79% of individuals engaged in gaming and 47% in gambling [41]. Around a quarter of university students were suggested to fall between moderate to high risk for their gambling behaviour, with potential implications on future gambling behaviour being problematic [41]. Similarly, over 90% of university students reported playing video games [42]. Many games share features and mechanics related to gambling, such as virtual currency and betting/wagering [43]. This highlights a key concern relating to the potential overlap between gaming and gambling. To demonstrate, ‘Whales’ is the term used by the gambling industry to describe someone who is a regular casino gambler with a high-income [44]. This concept has been applied to free-to-play games to describe a player who spends a lot of money quickly to progress in the game which has been suggested to apply to around 10% of players [45, 46].

Loot boxes have been an especially problematic feature for children and young people, with reports of some individuals spending considerable amounts of money [47]. An article by the British Broadcasting Corporation (BBC) highlighted that young people and vulnerable adults were spending hundreds to thousands of GBP on in-game purchases including Electronic Arts (EA) sports games [48]. The management of EA have suggested that loot boxes are ethical and create fun, in what can be likened to a Kinder Surprise Egg [49]. However, this conflicts with previous research which found that loot boxes are related to problem gambling [48]. For example, Delfabbro and King [50] the theory of gaming leading into gambling (known as the *gateway hypothesis*) and suggested more qualitative research is needed to support understanding this potential overlap. Particularly, the implications for long term outcomes, for example, would those that engage with loot boxes then get into gambling later as a result.

### Gambling policy

Concerning the gaming aspects of the policy (not specifically related to video or digital gaming), the UK Gambling Act [51] highlighted the role of the chance for a prize as essential criteria to define what gambling is. Griffiths [8] suggested that loot boxes are virtual games of chance and Xiao [32] suggested they are randomised in-game rewards of varying in-game and/or real life ‘value’. Parke and Griffiths [52] highlighted the role of rewards and near misses in reinforcing gambling addiction and the pursuit of winning, in line with behaviourist theories. With concerns of loot boxes normalising gambling behaviour [32], this can also be applied to gaming, specifically loot boxes wherein the player may feel that they come close to winning a desirable item or find the process of opening the boxes and receiving prizes as rewarding. Zendle and Cairns [47], in a large-scale survey with 7422 gamers, found that more money spent on loot boxes was associated with greater severity of problem gambling traits. Furthermore, near misses, in-game currency and paying for loot boxes enhanced the relationship with problem gambling and buying loot boxes [53], the relationship was found again in a later replication study [54]. This has further implications when connecting to gambling as it means there could be some similarities with the design features of loot boxes (such as the in-game advantages and near misses), but without some of the traditional gambling features (such as the cashing out). Therefore, due to the similarities with gambling and the potential for gambling related harms, this highlights the need for more and specific regulation of loot boxes, including ethical design considerations [39].

The UK Digital, Culture, Media and Sport Committee parliamentary meeting [55] debated the role of loot boxes in free-to-play games as a form of ‘game of chance’, and the redefinition of loot boxes as a form of gambling in which FIFA [33] were used as an example. One of the issues around loot boxes and gaming is the relation between in-game spend and monetary value after purchases. Most games have a one-way system where in-game items and currency do not relate to real-life money/currency (including secondary markets, where items can be resold/exchanged). The secondary markets can support the monetary value and selling of in-game purchases and/or accounts. In FIFA19 [55], the odds of obtaining certain cards for the chance of winning certain in-game FIFA players were reported in less than 1% of packs [56]. The gambling regulators in the Netherlands required the cashing out feature in games to be disabled as well as fining EA for being in breach of this on their FIFA games, whereas the UK (which has similar legislation) took a softer approach [39]. This raises an important question of whether this is a form of gambling and highlights the need of transparency in the disclosure of probabilities and items which can be obtained [e.g. 17].

### Related work

Papagiannidis et al. [12] highlight the role of real and virtual money in games, specifically the role of virtual assets, including how real money is used and invested in virtual assets such as items for avatars (e.g. Second Life [57]). Jernström [46] observes how free-to-play games are driven by in-game purchases, which can create ethical concerns about how players are being motivated to make these purchases. It has been suggested that the main motivations for in-game purchases are related to social appeal, exclusivity, function, and collectability [58] but can also be due to impulsivity [59]. Six factors have also been found to be related to in-game purchases: Unobstructed play, Social interaction, Economic rationale, Competition, Indulging children, and Unlocking content; with the first three factors in particular being specifically related to amount of in-game spending [60]. Since loot boxes involve in-game transactions there is scope to research loot boxes further and to examine players experiences and perceptions of loot boxes.

King et al. [43] suggested a checklist of features for understanding how much a game relates to gambling (See Table 1) but comparisons and conceptualisation of loot boxes with gambling are still emerging [e.g. 9]. More research could support to the conceptualisation of loot boxes from participants experiences which can aid drawing parallels with gambling. The concern of loot boxes creating a parallel with gambling has been previously highlighted [61, 62] and recent quantitative/mixed methods research has suggested an overlap between loot boxes and gambling where most participants saw loot boxes as gambling or related to gambling behaviour [63, 64]. Nicklin, et al., [64] using mixed methods explored the motivations of participants from the general population purchasing loot boxes and identified that motivations were related to the following themes: opening loot boxes, value of the content, game motivations, social influence, emotional/impulsive, and Fear Of Missing Out (FOMO) as well as connections between motivations to buy and signs of problematic/ harmful behaviour. The research focused on different motivations to buy loot boxes, but the game mechanic of loot boxes and how players experience loot boxes as a feature of a game remain underexplored and unclear. Although the research recruited a wide age range of 20–56, more research is required to address the potential age differences and experiences with loot boxes. For instance, research suggests that university students have previously been highlighted as group which could be vulnerable to gaming and gambling related harms [11, 41, 65]. Hence, the focus of this research was with young people at university. Additionally, the authors report stakeholders were included in the research as participants, some of which reported having a professional connection to loot boxes which may have caused a bias in the results [64]. Another qualitative study into the motivations of purchasing loot boxes suggested that they were brought for entertainment and socialising but were related to impulsivity and distorted beliefs from the player and were deemed to be an in-game feature rather than gambling [59]. Further qualitative research is therefore needed to explore this topic in more depth and understand conceptualisation of loot boxes from participants as a gaming feature (the experience of this gaming feature) as well as the potential parallels with gambling from a specific sample/group.

**Table 1:** King et al. [43] description, definition, and examples of gambling features in games.

Features	Example and definition
**Interactivity**	Relates to the players’ involvement (passive or active)
**Monetisation level**	Financial elements of play
**Betting/wagering mechanics**	If players are able to make bets and stake items
**Determination of outcome**	Skill vs. luck
**Measurement of outcome**	Financial and non-financial outcomes (e.g. progression in-game, in-game items)
**Structural fidelity**	The extent it represents traditional gambling activity
**Context**	Where does this activity fit within the game (e.g. stand-alone, mini or external)?
**Centrality**	How much focus is on the gambling activity within the game (e.g. primary, secondary mandatory or optional)?
**Advertising**	the level of advertising included

### Rationale of the present study

Given the growing concerns over gaming and loot boxes in particular, more research is needed to understand this game mechanic and how it relates to in-game, real-life experiences, and gambling behaviour. Furthermore, the on-going debate within the media, industry, policymaking bodies, and government highlights the importance and need for participatory action research from the players’ point of view into exploring loot boxes conceptually, and if there is a perceived overlap with gambling. It is proposed that to consider the role of loot boxes and gambling from the players’ point of view, we first need to define this game mechanic/feature from insights and experiences of players to understand how they conceptualise it. This will help understand the potential comparisons and overlaps with gambling. A purely qualitative approach enables a participatory action research design through exploring the insights of players and their experiences of loot boxes, and gambling in the UK from young people. The research aims to answer the following questions:

RQ1 What are the players’ perceptions and experiences of loot boxes?
RQ2 How do loot boxes compare with gambling from the players’ perspective?

## Materials and methods

### Participants and procedure

The study comprised a purposive convenience/voluntary sample of 21 University students (14 males, 4 females, 3 unknown) aged 18–27 (*M*_age_ = 21.55, *SD* = 1.99) who took part in either a structured interview (*n* = 13) or an open-ended online survey (*n* = 8). No participant drop-out or withdrawal took place. This demographic was appropriate due to the popularity of gaming and gambling among this age group [41] and knowledge of loot boxes in games. An age restriction of above 18 was selected for this study; in the UK the legal age for the majority of gambling activities is 18. Therefore, participants under 18 were excluded from the study due to the content of the interviews discussing topics related to gambling [47]. Participants were recruited via an advertisement on social media within the University’s gaming society forum Facebook. This forum was appropriate as members were assumed to play video games, identified as gamers, and have some knowledge of loot boxes. Participants were included if they had previous experience with video games and would be happy to discuss loot boxes. Participants were excluded if they did not play video games or felt they were unable to contribute to the topic of loot boxes. Hence, targeting the universities gaming society would support participants meeting the inclusion criteria for study. Participants were provided with an information sheet about the study and a consent form to sign if they agreed to take part (written consent). Data collection took place between January and April 2019.

All participants were asked 19 open and closed questions (designed for the purposes of this study for one schedule) relating to their experiences of loot boxes and their perceptions of loot boxes as a type of gambling. This included questions about their gameplay (genre and platform), familiarity and experiences with loot boxes, and whether they had ever purchased loot boxes with real money (S1 File). Interviews took around 30 minutes and were conducted face-to-face. For eight participants who could not be interviewed face-to-face (due to undisclosed reasons), the same questions were presented in an online open-ended survey. At the end of the study, participants were debriefed (including a reminder of withdrawal procedures) and thanked for their participation. Data saturation was supported by using interviews and open-ended questions to gather both depth and richness in the data as well as having a sample size larger than 6 [66]. Ethics approval was obtained via the University Faculty of Science and Technology Ethics Committee. Interview responses were audio recorded through an audio recorder and then transcribed. During transcription all responses were anonymised, and participants’ responses were assigned a participant code. Then the data was processed into a word document for analysis to take place. No field notes were made during or after the interviews.

### Theoretical approach and data analysis

Interviews and responses were transcribed and analysed by two researchers using Thematic Analysis [67, 68]. Thematic Analysis was deemed appropriate given the requirement to explore overarching themes from the data and to investigate participants’ experiences with loot boxes. Exploring the data with two research questions allowed us to 1) take a neutral stance in exploring loot boxes and the concept itself, and 2) draw parallels with gambling after clarifying the nature of loot boxes. This two-staged process allowed findings to overcome any possibility of research bias when investigating the parallels between loot boxes and gambling. As such applying the research paradigm of Constructivism; how individuals build their knowledge and understanding of ideas and therefore experiences which is fitting with the research question to participants experiences and conceptualisation of loot boxes [69].

#### Reflexivity and enhancing trustworthiness

Following suggestions by Braun and Clarke [70], a process of reflexivity was acknowledged to facilitate better Thematic Analysis, given that the researchers had different gaming and loot boxes experiences and were from different disciplines (Computing and Informatics, Psychology). Interview and survey questions and the view of loot boxes design and algorithms were provided by the researcher from the Computing and Informatics department, whereas analyses and knowledge of human-computer interactions were offered by the researchers from the Psychology department. This collaboration allowed for reflexive commentary [71] during the data analysis stage. The discussion on Reflexivity is continued in the limitations.

## Results

During the analysis, weekly meetings were arranged to offer reflexive commentary on the themes and their interpretation [70], to add rigour to the process and encourage deliberate and reflexive engagement with the six steps. Step 1, the interviews were transcribed, collated, and read through several times. Step 2, initial codes for RQ1 were systematically developed and discussed as a group, with considerations to ensure that all data was included equally and was developed from participants’ responses to avoid researcher bias. Step 3, these codes were further grouped and clustered into themes and subthemes. Three distinct themes were developed with supporting quotes. These themes were then checked with the other themes and subthemes. Step 4, these themes were reviewed and discussed which led to the creation of a thematic map. During this stage, two additional subthemes of ‘Social’ and was created and added to the ‘Implementation’ theme. Theme extraction was checked to ensure they are exclusive, and no further themes can be developed. Step 5, themes were defined and named for RQ1. These themes were developed further and reviewed for RQ2 through regrouping into overarching themes Re-grouping RQ2 specific quotes that related to gambling, the player, and game design were highlighted. Step 6, the themes of RQ1 and RQ2 were analysed and described in the results section [67]. Data analysis took place between May and August 2019.

For questions on the gameplay and familiarity with loot boxes, most participants reported a “good” or “great” understanding of loot boxes (81%), with the remaining 19% reporting an “OK” understanding of loot boxes. Most participants (52%) reported that they had purchased loot boxes using real money and had indifferent (32%) or bad (23%) experiences with loot boxes. Most participants played video games on a console/platform (i.e. Nintendo DS, PlayStation Vita, PlayStation 4, Nintendo Switch; 48%), a PC (42%), or a mobile device (10%). The two most frequent genres of games played by participants were Shooters and Role-Playing Games (RPGs) video games. The most mentioned game/scenario with loot boxes was Overwatch [19].

### Themes of RQ1: What are the players perceptions and experiences of loot boxes?

**Fig 2:**
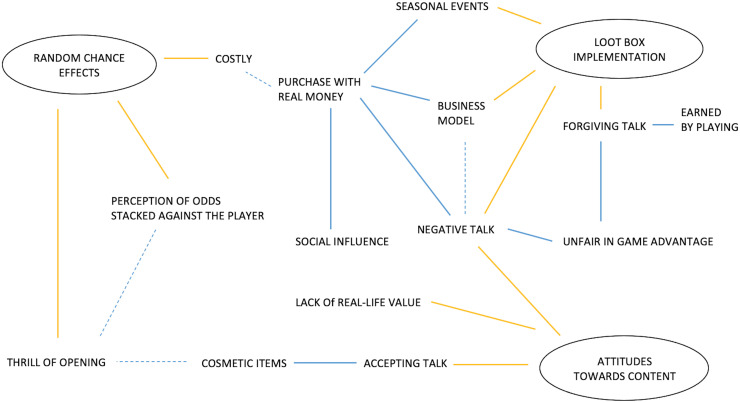
Thematic map of participant’s conceptualisations of loot boxes.

Fig 2 shows the thematic map of themes and sub-themes which emerged from the data. The ovals represent the themes, with the yellow lines linking the theme to its main sub-themes: blue solid lines represent direct relationships, and the blue dotted lines represent in-direct relationships.

#### Random chance effects

The first theme ‘Random chance effects’ represents the participants’ experiences of random outcomes of loot boxes and consisted of 3 sub-themes: *Perception of odds stacked against the player (POSAP)*, *Costly*, and *Thrill of opening (TOO)*. *POSAP* refers to the odds that participants feel are stacked against them and the unlikelihood of winning what they wanted. A sample quote is: “There’re such low odds to get anything you want”. This sub-theme is also associated with the sub-theme *Costly*, refers to the perceived consequences of chance represented by spending more on loot boxes to overcome the randomness of typical loot boxes. A sample quote is: “…if you see something you want and you can’t get it any other way, the odds of getting it are too low”. *TOO* refers to the specific feelings experienced with regards to the rewards and thrills at the moment of opening loot boxes. A sample quote is: “You know there is a bit of a thrill to it”.

#### Implementation

The second theme ‘Implementation’ represents the environment and context that loot boxes mechanics are presented to the player and consisted of 5 sub-themes: *Seasonal events*, *Business model*, *Negative talk*, *Forgiving talk*, *and Social influence*. *Seasonal events* refer to the exclusivity and time limitations imposed on purchases of loot boxes. A sample quote is: “Overwatch, when like the holiday skins come out and you got a limited amount of time, so you have to get the loot boxes in that time frame…”. *Business model* refers to the relationship between loot box purchase, loot box mechanics, and how loot boxes were applied in games. A sample quote is: “[loot boxes] lead developers to a cash grab instead of a good game”. In relation to this, some participants felt they were deliberately slowed down from progressing in the game and having to play repetitive or boring levels to encourage them to purchase loot boxes to progress faster. *Negative talk* refers to the thoughts of additional costs after already purchasing the game and the implications for developers being more money focused rather than play focused. A sample quote is: “[loot boxes] lead developers to a cash grab instead of a good game”. *Forgiving talk* refers to the perception that loot boxes were more fitting to free-to-play games and/or the role of creating loot boxes to be earned only through gameplay, rather just the paid option. A sample quote is: “Yes, I much prefer a system where loot boxes are only attainable through playing, provided the developer doesn’t make it incredibly grindy [difficult]”. Finally, *Social influence* refers to players’ experiences of the social factors involved in buying loot boxes, which comprised two aspects: purchasing loot boxes as they saw others doing the same despite claiming it did not influence them (“I am not influenced by the purchasing habits of others”), and purchasing loot boxes due to the effect of social influences (“…youtubers that do that mystery box thing and telling their viewers to get their parent’s credit cards, which is the worst thing to say to someone”).

#### Attitudes towards content

The third theme ‘Attitudes towards content,’ represents players’ feelings and thoughts towards the contents of loot boxes and consisted of four subthemes: *Accepting talk towards*, *Cosmetic items*, *Negative talk*, *and Lack of real-life value (LORLV)*. *Accepting talk* refers to feelings about items obtained through loot boxes that aided progression in the game or a cosmetic change. *Cosmetic items* refer to the specific in-game items that were discussed, and were viewed more positively by participants, a sample quote is: “I was alright with Overwatch loot boxes because they are mostly cosmetic, so you don’t have to even come in contact with them at all”. *Negative talk* refers to negative views about loot boxes and the game itself when it only provides advantages for those who pay, as some individuals may pay more than others. A sample quote is: “If you can’t afford them, you as a player feel cheated out because other players will have an advantage over you”. Finally, *LORLV* refers to perceptions and experiences of low or little real-life monetary value of the purchases from loot boxes. A sample quote is: “…the difference between a loot box in a game and a card pack in real life is that you’re actually getting something physical and tangible. And worst-case scenario you get a card you didn’t want but it’s rare, you can sell that”.

From the analysis, Table 2 suggested how loot boxes are the secondary focus, in that players will want to play the game rather than specifically play for the loot boxes.

**Table 2:** The application of loot boxes and the results from this research to King et al. [43] description, definition, and examples of gambling features in games.

Features	Example and definition	Applied to loot boxes from the results of this research
**Interactivity**	Relates to the players’ involvement (passive or active)	The player is active in the buying and opening but can range in the level of activity for the contents and probability
**Monetisation level**	Financial elements of play	Vary as loot boxes can be purchased for virtual/in-game or real currency or free (but tend to be a tedious process to get them)
**Betting/wagering mechanics**	If players are able to make bets and stake items	Many do not have this feature
**Determination of outcome**	Skill vs. luck	The majority seem to be luck/probability-based that is programmed through algorithms but the how random and the algorithms used are not clear.
**Measurement of outcome**	Financial and non-financial outcomes (e.g. progression in-game, in-game items)	In-game items or progression in the game. Secondary markets to sell in-game items
**Structural fidelity**	The extent it represents traditional gambling activity	It can be compared to raffles, lotteries, toy machines.
**Context**	Where does this activity fit within the game (e.g. stand-alone, mini or external)?	It is game feature as a mini-game within the main game
**Centrality**	How much focus is on the gambling activity within the game (e.g. primary, secondary mandatory or optional)	Secondary but most are optional (e.g. given for free can choose not to open them in most cases)
**Advertising**	Is/the level of advertising included	Some contain adverting e.g. Twitch prime with Overwatch. Amazon for Hearthstone.

### Themes of RQ2: How does loot boxes compare with gambling from the players perspective?

**Fig 3:**
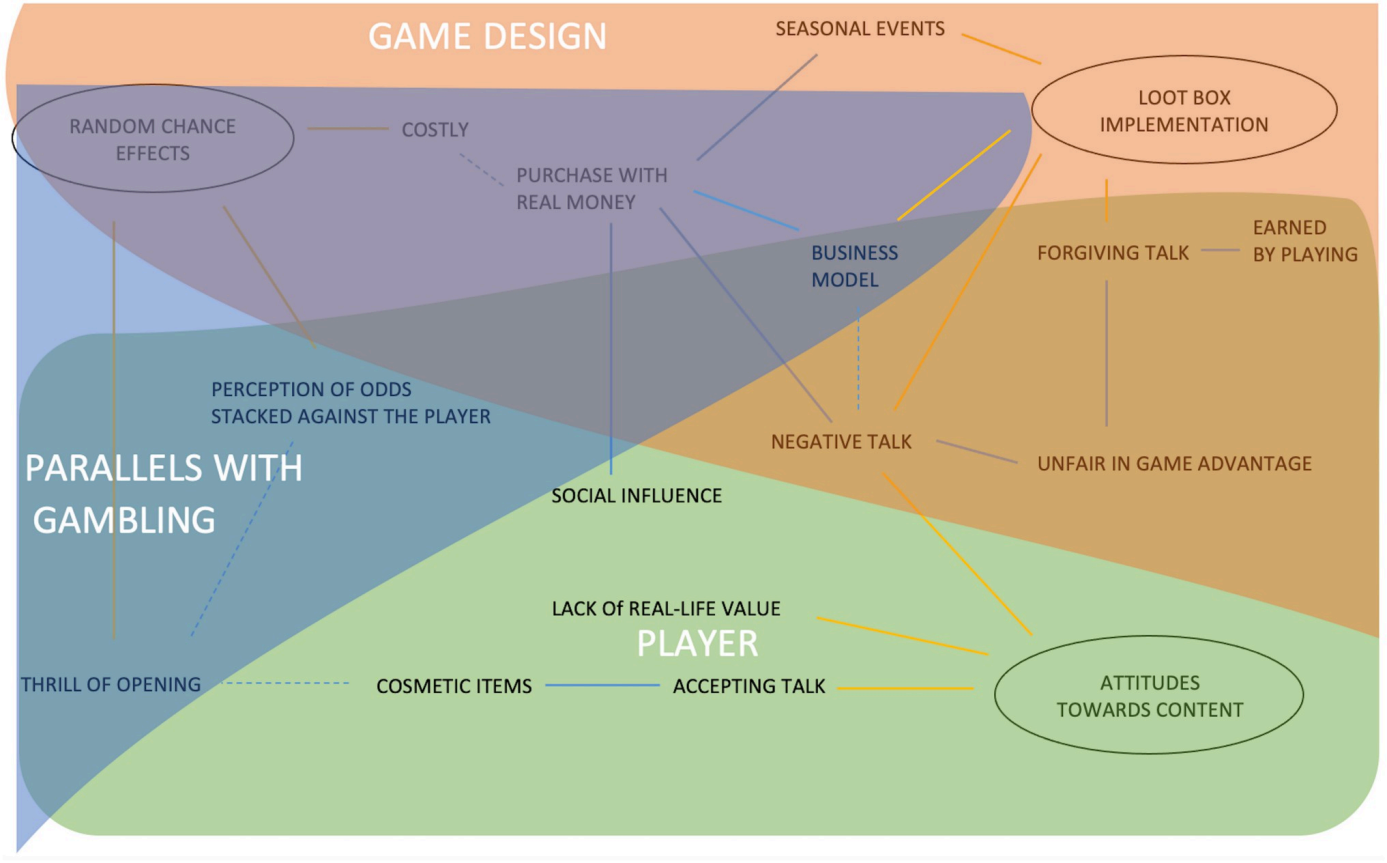
Represents the adapted thematic map from RQ1. Game design themes are grouped in orange, parallels with gambling themes are highlighted in blue and player themes are highlighted in green for RQ2.

After analysing the data to explore what loot boxes mean from the players perspective, the data were further analysed to explore if a relationship between gambling and loot boxes was reported. The three main themes from RQ1 were suggested to represent three aspects of the players’ experiences with loot boxes, the parallels with gambling, game design and player (Fig 3). Fig 3 shows the themes of parallels with gambling, game design, and the player, all are colour coded with their subthemes. Note, that the subthemes of the perceptions of odds being stacked against the player and business model were connected to all three themes.

#### Parallels with gambling

The parallels with gambling were represented by the theme ‘Random chance effects’ and its sub-themes (*POSAP*, *Costly*, *TOO*, *Business Model*). These were suggested to have a direct link to gambling. *POSAP* related to parallels of odds stacked up against players with loot boxes and gambling activities. A sample quote is: “They are basically gambling the house always wins is a saying for a reason”. *Costly* referred to players spending more and more on items they desired, which was compared with gambling. A sample quote is: “just like gambling, it can ruin you financially”. *TOO* referred to players’ feelings and experiences of opening loot boxes; like that of winning in gambling. A sample quote is: “…gambling, at the end of the day you spin, take for example slots, you spin 3 little tiles and you’re hoping to see 777 appear until you get the jackpot”. *Business Model* related to how application of loot boxes can feel like gambling for the player. A sample quote is: “Definitely not a fan of that, if they end up doing it like this it will end up being basically like gambling [on games promoting loot boxes purchase].”

#### Game design

Game design was by presented by the themes of ‘Implementation’ and ‘Random Chance Effects’ which represents how loot boxes are incorporated in games and link to purchase intentions, with the following sub-themes: *Seasonal events*, *Business model*, *Costly (Purchase with Real Money)*, *Forgiving talk*, *Earned by Playing*, *Negative talk*, *Unfair in game advantage*. Examples for *seasonal events* and *Business model*: *Seasonal events* represented experiences of Fear of Missing Out (FOMO) on loot boxes purchase deals and influences on spending real-life money on loot boxes. A sample quote is: “Overwatch, when like the holiday skins come out and you got a limited amount of time, so you have to get the loot boxes in that time frame and then it’s like the day before the event ends and you really want that ‘Tacer’ [sic] skin so you’re actually spending real money on loot boxes”. *Business model* represented the relationship between loot box purchase, loot box mechanics, and how loot boxes were applied in games. *Business model* was suggested to be connected to gambling as game design can influence purchase intentions. A sample quote it: “[loot boxes] lead developers to a cash grab instead of a good game”. In relation to this, some participants felt they were deliberately slowed down from progressing in the game and having to play repetitive or boring levels to encourage them to purchase loot boxes to progress faster. See S1 Table for more examples.

#### Player

The theme ‘Player’ represents the factors relating to the willingness and attitude towards buying loot boxes and consisted of the following sub-themes: *Cosmetic items*, *Social influence*, *Accepting Talk*, *TOO*, *LORLV*, *Forgiving talk*, *Earned by playing*, *Negative talk*, *POSAP*, *Business model and Unfair in game advantage*. Examples for *Cosmetic items*, *Social influence*: *Cosmetic items* represented purchase decisions of loot boxes based on their appeal. A sample quote is: “I have purchased loot boxes in several games, but only those that provide aesthetic changes”. *Social influence* was moved from ‘Implementation’ to this theme and refers to players behaviour and experiences, including how they perceive themselves not influenced by if others are buying loot boxes but suggest others are prone to be influenced by purchasing habits of others, suggesting a social influence to buy Loot boxes. A sample quote is: “… [loot box practice] probably teaches gambling characteristics” (S1 Table for more examples).

## Discussion

Using participatory action research, this study explored the insight of players and their experiences of loot boxes within video games to investigate any parallels with gambling. The analysis for RQ1, relating to the players perceptions and experiences of loot boxes, identified three main themes: Random chance effects, Attitudes from content, and Implementation. Players experiences and perceptions of loot boxes were suggested to be more negative highlighted from the subthemes such as Costly, Negative talk, Perception of odds stacked against them, Negative talk towards in-game advantage, and Lack of real-life value (see Figs 2 and 3). These findings echo previous qualitative and mixed-methods research [59, 64]. Building on previous research, the results from RQ2 highlight the game mechanics/features as well as the interaction that takes place from the suggested overarching themes of ‘Parallels with Gambling’, ‘Game Design’, and the ‘Player’. Loot boxes comparisons with gambling from the players perspective **s**uggested that most participants perceived loot boxes as gambling, similar to previous research [e.g. 63, 64], but that this relationship was both an indirect and direct parallel to gambling (see Table 2). The complexity of the relationships of themes were represented from the two research questions, highlighting number of themes and their relationship with each other. Thus, exploring loot boxes for RQ1 provided good foundations for RQ2 and helped reveal the overarching themes.

### Social and cosmetic factors in loot boxes

Social factors were suggested by participants to play an important role in relation to the purchase of loot boxes, in line with Nguyen et al. [59]. Previous research has identified that the majority of the microtransactions in gaming in the last 10 years were related to an increase in cosmetic factors [72]. The Children’s commission report [73] highlighted how the default settings in games, including the Skins (the cosmetic appearance), were seen as less valuable as well as the perception of other players viewing it as less valuable in that the player cannot afford the upgrades. Banks and Bowman [74] found the social prominence of avatars in Massively Multiplayer Online games (MMOs) and these interactions can vary from social to para-social; which could also relate to why cosmetic items for avatars were seen more positively by participants as well as demonstrating the complex social environment of gaming. Participants also highlighted the role of social influence but suggested they were not as susceptible to this like others, but it is acknowledged this is most likely a bias [75].

Previous research has suggested a prominence of social factors in gaming such as social identity [76] and the Proteus effect referring to how the features of the avatars lead to change in the player behaviour [77]. Plus, players who purchase these in-game upgrades have been found to be viewed more negatively by other players in multiplayer games [10], a finding we also reported. The consequences of such could be attributed to empowering in an unfair manner and ruining the spirit of gaming and was represented by players’ negative views about the gaming industry and their in-game playing experiences. Participants also alluded to the role of FOMO and related it to the theme of seasonal events. This has important implications for game designers as well as the players, in that loot boxes should not be designed to manipulate or persuade players into buying. For example, suggested leaked documents of game designers using AI to influence microtransactions and purchasing behaviour [78]. The role of game design was highlighted through the theme of the Business model, previous research found a small connection between loot boxes and free–to-play and shooter games [79]. Drawing the two concepts together of purchasing and business model, a news article suggested that FIFA [33] make around a third of their profit from loot boxes [80]. More recently there have been examples of gaming companies facing legal action around loot boxes [81].

### Parallels with gambling

The parallels with gambling could demonstrate the role of technology specifically evolving and creating hybrid gambling experiences of real-life gambling. King and Delfabbro [11] highlighted the hybrid nature of gaming and gambling with the developments of technology. While loot boxes may not share all the components of traditional gambling, it was suggested to share some characteristics, particularly the role of Random chance; which previously been highlighted in previous research by Griffiths [8] describing loot boxes as virtual games of chance, and policy discussions [51, 55]. Explanations of gambling and gaming behaviour have been related to behaviourist theories of rewards [52]. It seems from our exploration that the random and chance elements were related to the rewarding feeling and the rush from opening loot boxes, as well as the perception of the odds being stacked against them. Lack of value could translate to a form of loss as the new item may be unwanted or lack in-game value. This also relates to King et al.’s [43] checklist of features of gambling in gaming, which showed the potential of loot boxes to be gambling. Furthermore, these parallels could have the potential to act as risk factors for purchasing loot boxes and gambling. This could be demonstrated from loot boxes potentially relating to traditional forms of gambling but applied in a virtual form, and further benefiting from player data and highlight the need for a policy of loot boxes functions in games (e.g. the total amount of money that can be spent).

The overarching themes of ‘Player’ and ‘Game design’ highlight another key aspect is the interactivity between the player and the game [82, 83]. This interactivity was also highlighted by King et al. [43] with the checklist of gambling behaviours in games. Therefore, interactivity could be a key factor for the relationship between implementation/games design and players perceptions. This is important as it has implications for players and the gaming industry; including whether rating systems should include loot boxes as gambling to protect young people. PEGI (Pan European Game Information) [84] currently includes a label for gambling content and the ESRB (Entertainment Software Rating Board) [85] website allows for gambling content in games to be specifically searched. Zendle et al. [53] urged the video game rating systems to include loot boxes to support the communicate of game content. PEGI [84] has since added an additional label of ‘Includes Random Items’ [32]. While these labels aim to help inform users of content, they have been criticised for not supporting the user. Thus, more criteria and detail may need to be added to these descriptions [86].

Arguably, even if loot boxes are a secondary aspect to video games, they could be programmed to exploit players through the gameplay/behavioural data collected rather than being programmed to be random. As such, this has implications for potential gambling aspects. Zendle and Carins [47] recommended that the game age rating boards change loot boxes to over 18 to be consistent with other gambling behaviour support reducing gambling-related harm. Furthermore, over 90% of mobile games containing loot boxes are currently rated a 12 plus, which has further repercussions for young people encountering them [14]. Some companies such as EA games have already been receiving negative media attention in that regard [48, 49]. King and Delfabbro [87] highlighted the suggestions of social responsibility for game design and purchasing through transparency, consumer protection, information, and industry accountability. This could then support users not getting into financial difficulties and making positive purchasing decisions.

### Supporting the player

To help reduce the potential financial harm of loot boxes and in-game transactions, one recommendation could be to change the way these transactions are processed. News articles have suggested that loot boxes should be banned for children and called for in-game spending to be regulated [88–90] with the UK House of Lords report [91] calling for immediate action on loot boxes to support safeguarding this age group. Gambling transactions are specified in banking statements; the same could be done for in-game purchases in loot boxes to support the user. Furthermore, additional policy regulations and guidelines are needed to support many users (not just minors) who purchase and interact with loot boxes (e.g. regulations on card packs, [92], avoiding predatory design [18–20, 38]). Some participants discussed the role of addiction especially for other players. Unlike other addictive behaviours, such as substance addiction, gambling and gaming are classed as behavioural addictions with the role of repetitive behaviours being specifically highlighted [93]. The Diagnostic and Statistical Manual of Mental Disorders (DSM-V) specified that Gambling Disorder (GD) needs to contain at least four components from the of behaviours listed (excitement from increasing money used in betting, negative emotional responses to cutting down, and chasing losses) [94, 95]. From our findings, loot boxes seem to relate to the following behaviours mentioned previously: spending for excitement, negative feelings, participants reporting others may find it difficult to stop, and could be chasing items; suggesting a potential for loot boxes matching gambling disorder. Alternatively, recent research suggested a connection between the amount spent on loot boxes and problem gambling even when the cashing out feature is removed [53].

These results also connect to the characteristics of behavioural addiction [96]; loot boxes could relate to Mood Modification as they are suggested to provide thrills and excitement. Conflict may also, as participants may not like them or want to spend money but feel they have to. Tolerance could be represented by the acceptance some of the context around loot boxes, which could encourage further purchasing. Withdrawal could be represented by participants rationalising their experiences in the interviews and the negativity that was reported in the themes. Relapse was not represented as directly as the other factors, but it could be suggested that loot boxes could still be bought even if players did not want to. Future research could explore these behavioural factors in loot boxes further, especially in relation to the potential role of relapse behaviours. Support for this comes from recent research which suggested that problem gamblers who played the game Heroes of the Storm [97] spent less money in video games when loot boxes were removed [72].

Currently, more young people play video games than they do gamble but there are concerns for the potential risks for young players of these games [41]. Our results find that there can be other risk factors that need consideration as loot boxes were suggested to share features of gambling, but questions remain to the consequences of long-term exposure to loot boxes and online/virtual gambling. This could be likened to drug-taking chronic exposure to drugs has been found to increases the likelihood of drug addiction [98] and that some milder drugs can act as a gateway for harder drugs [99] and often there are more than one/multiple drug addictions [93]. Although Marks [93] suggested that multiple behavioural addictions are not necessarily as common as substance addiction; it could be that technology is creating and/or tapping into the same behavioural pathways for the addictions and that they may transfer between platforms.

Specifically, there are implications for how technology develops, how loot boxes operate in games, and the overlap that this may have with gambling. For example, King and Delfabbro [11] suggested that technology developments have led to simulated gambling (i.e. gambling through technology with no monetary features such as free-to-play online casinos). The gaming industry even compares free-to-play games to the Japan *Gacha* toy machines where a random toy is produced in exchange for money [46, 100]. However, there is a debate around whether we can consider simulated gambling as gambling. This highlights the many different forms of gaming and gambling that exist and poses several questions: i) What counts as gambling? ii) When does it become harmful? and iii) What is the boundary between virtual and real? For example, is GTA’s Diamond Casino and Resort [28] closer to real-life gambling than simulated gambling as players can use in-game currency within a virtual casino? Furthermore, some of the advertising for games has been suggested to be relating to gambling activities such as NBA 2K20 [101, 102], suggesting it is not just gaming content but how games are advertised. Xiao and Henderson [103] stress the application of ethical game design frameworks for loot boxes to support the users, hence highlighting the importance of the considerations needed when designing this game feature.

Previous research has found specific behavioural types for social media use [104] and there could also be behavioural types for gaming and gambling (e.g. the content of the games played could be essential to understanding the extent to which the player experiences gambling related features). It is important to consider that this potential transfer of problematic behaviours on different platforms could be due to some individuals being more at risk than others of excessive and addictive use, (e.g. personality factors and co-morbidities) [105]. However, Tamborini [106, 107] suggested that the type and content of media including video games may have long term influences on the selection of media and could be applied for virtual gambling behaviours in that they become salient and desired. Hence, it is important to consider the overlaps with purchasing and in-game decision making in that loot boxes may become normalised through their decision making and this connects to content of the games sought out by the individual.

### Limitations

Our findings are derived from structured interviews and open-ended online surveys, which provided a rich array of perspectives of loot boxes and gambling. Yet, the study had limitations. First, it is possible that participants provided socially desirable answers that reflect how loot boxes are portrayed in the media. As such, findings should be interpreted with caution. While social desirability biases are common in qualitative research [108] future gaming research could ameliorate their influence by adopting a mixed-methods approach that observes and interviews participants both during and immediately after interacting with game artefacts, such as loot boxes [109]. Second, participants’ existing knowledge of loot boxes could have influenced their answers regarding them being associated with shooter and free-to-play games [79]. Similarly, given that loot boxes are prevalent in all types and genres of video games [110, 111], their emergence within the game may also be influenced by whether they are perceived as a part of the game or as a source of gambling which in part explains the debates around if it related to gambling or not. It is also acknowledged that having a mixture of interviews and online surveys was a limitation to the study as responses may have been different between the methods. Finally, given that media coverage of the blurred boundaries between games and gambling activities are often context and country specific [112], our findings are limited in scope to those in the UK. Future research could look to explore perceptions of loot boxes in other countries.

### Future research

Whether loot boxes fulfil the requirements to be classified as gambling is a legal matter that will vary across countries. However, the findings presented here suggest that there is a relationship between loot boxes and gambling. As such, future research to investigate this relationship is warranted. Given that loot boxes are implemented in different types and genres of games, it would be beneficial to explore players’ perceptions of the emergence and design of loot boxes across different gameplay styles and genres. Although some research has explored the role of in-game purchases in Esports [79] and free-to-play [113] games, more research is needed to understand the design mechanics and motivations underpinning purchases of loot boxes. Future research could also aim to investigate the long-term implications of virtual gambling and gameplay. For example, the last few years has seen a surge in games utilising Virtual Reality (VR) technology due to its heightened sense of realism and promise for immersive experiences [114]. As such, VR offers the creation of novel consumption experiences [115, 116], which could ultimately influence how loot boxes are perceived and consumed by players. The potential impact of VR on loot boxes consumption is not yet known.

Finally, research should explore the role of transparency and fairness in relation to the advertisement strategies and actual content of loot boxes offered within games. For instance, the way in which new characters can be obtained in some loot boxes may blur the boundaries between content that is regarded as game progression or items that are avatar-specific [8]. Similarly, the purchase of random item loot boxes may deceive some players as they are not presented with information about their contents prior to the purchase. Investigating this overlap with consumer behaviour and decision-making within games may help to revise legislation aimed to protect users from purchases that would not be deemed acceptable in real life, such as the Consumer Protection from Unfair Trading Regulations and avoid predatory practices [18, 117].

## Conclusions

This study investigated experiences with and perceptions of loot boxes as elements of gameplay and source of gambling. Our thematic analysis identified three themes associated with loot box experiences: Random chance effects (i.e. perception of ‘arbitrary’ odds stacked against the player), Attitudes towards loot box contents (i.e. perception of real-life value and in-game advantages), and Implementation of loot boxes (i.e. environment and context in which loot boxes mechanics are presented). Further analyses revealed key overarching of themes to understand players’ experiences of loot boxes (i.e. accepting talk), game design (i.e. seasonal events), and parallels with gambling (i.e. random chance effects). Overall, it was suggested that loot boxes overlap with gambling and that new definitions of gambling are required as previous definitions are not enough to encompass virtual spaces and digital technologies and the hybrids they are creating.

## Supporting information

S1 File Interview questions and schedule. (DOCX)

S1 Table Theme table for RQ1 and RQ2. Theme table with sub-themes and supporting quotes for RQ1 and RQ2.
(DOCX)
