# Supplementary material for: What’s in the box? Exploring UK players’ experiences of loot boxes in games; the conceptualisation and parallels with gambling
Source: PLoS One. 2022 Feb 9;17(2):e0263567. doi: 10.1371/journal.pone.0263567 (PMC8827416; doi:10.1371/journal.pone.0263567)
Supplement: S1 File — (DOCX) [file pone.0263567.s001.docx]

**S1 File. Interview questions and schedule**

1. How old are you, and what do you study at university?
2. What platform do you play games on?
3. What genre of games do you play?
4. How much do you know about Loot Boxes?
5. Tell me about your experience with loot boxes.
6. Have you ever bought loot boxes with real money?
7. What do you think about the experience of buying them?
   1. [if the answer is no] Have you seen anyone buying loot boxes and what do you think of their experience?
8. Does your opinion on loot boxes change if the game offers no way to purchase them with real money?
9. When other players buy loot boxes, how does that influence your decision to follow suit?
10. How would you feel tempted to buy loot boxes?
11. What do you think of the idea that games design could influence players to purchase loot boxes?
12. How do you feel about physical blind boxes or card booster packs?
    1. [Blind boxes refers to a physical box or package in real life in which you purchases with only a vague understanding of what's inside (for example products relating to a franchise).
    2. Card booster packs refer to physical foil packaging in which contains a set number of cards to a game (Pokémon, Yu-gi-oh, magic the gathering) the number of cards inside will always be the same however the cards themselves are random]
13. What are your main criticisms to loot boxes?
14. What are the main improvements you like to see in future loot boxes?
15. Some people see a comparison between loot boxes and gambling. What is your opinion on this?
16. How do you think about a risk that the use of loot boxes can become addictive?
17. Can you think of the positive things associated with loot boxes in players experience, feelings, and real life?
18. Can you think of any harm associated with loot boxes whether personal, social, financial and wellbeing in general?
19. Finally, can you list 3 most common scenarios in which you used or saw others use loot boxes?
