# Supplementary material for: What’s in the box? Exploring UK players’ experiences of loot boxes in games; the conceptualisation and parallels with gambling
Source: PLoS One. 2022 Feb 9;17(2):e0263567. doi: 10.1371/journal.pone.0263567 (PMC8827416; doi:10.1371/journal.pone.0263567)
Supplement: S1 Table — Theme table with sub-themes and supporting quotes for RQ1 and RQ2. (DOCX) [file pone.0263567.s002.docx]

**S1 Table. Theme table for RQ1 and RQ2. Theme table with sub-themes and supporting quotes for RQ1 and RQ2.**

| Theme RQ1 | Sub-theme | Quotes | Theme RQ2 |
| --- | --- | --- | --- |
| Random chance effects | Costly | “There's a chance you can spend £20 on a skin where you only spend £6 for”  “I’ll buy 5 orbs or something on Fire Emblem Heroes, and do a snipe, and not get what I want, and it is only £2 to get a few more. It is very easy to get into that cycle”  “The only thing with loot boxes that I have experienced people spend a lot of money trying to get the skins they want”  “Hearthstone [online card game where cards are earned via lootboxes] is just very much you need to spend more money to get good cards” | Parallels with Gambling and Game Design |
|  | Perception of odds stacked against the player | “There’re such low odds to get anything you want”  “They are basically gambling, "the house always wins" is a saying for a reason”  “…if you see something you want and you can't get it any other way, the odds of getting it are too low”  “The main problem with loot boxes in the beginning you have to buy a million loot boxes to get the items you need”  “Increase the chances for nicer items so loot boxes feel more worthwhile [when asked about how lootboxes could be improved]” | Parallels with Gambling and Player |
|  | Thrill of opening | “…and I like the thrill and rush of opening packs”  “You know there is a bit of a thrill to it”  “It does give you a bit of a rush”  “Definitely the feeling you get when you get something amazing”  “It feels really good when you receive a good item, however the negative feelings outweigh the good. | Parallels with Gambling and Player |
| Loot box Implementation | Seasonal events | “Yeah, the timed exclusives make me really most tempted [to purchase lootboxes]”  “You can unlock them [lootboxes] without buying them, but obviously you have to play a lot in order to do it. But because it's Halloween and it's a special event only going on for a week you have to play almost 24/7 for that whole week to be able to accumulate decent amount [of in game currency] to get enough loot boxes of the Halloween special events.”  “…then an event will roll around which is even better than the last one, so you’ll feel like you’ve wasted your money”  “Overwatch, when like the holiday skins come out and you got a limited amount of time, so you have to get the loot boxes in that time frame and then it's like the day before the event ends and you really want that Tacer skin so you’re actually spending real money on loot boxes” | Game Design |
|  | Negative talk | “Once I've paid £60 for a game, I don't want to spend money on top of it”  “If they are premium price, there's really no excuse to use them [lootboxes] honestly, they [game developers] are making enough money”  “[loot boxes] lead developers to making a cash grab instead of a good game”  “I despise the loot box practice, not so much because it milks the players' wallets, but because the whole paradigm has made games shallower”  “I think loot boxes paid for with real money should only be in free to play games/mobile games as a way for earning money for the developers” | Game Design and Player |
|  | Forgiving talk | “I think loot boxes paid for with real money should only be in free to play games/mobile games as a way for earning money for the developers”  “…if their like free to play games, then maybe [when asked about attitudes towards games using lootboxes]”  “Yes, I much prefer a system where loot boxes are only atainable through playing, provided the developer doesn't make it incredibly grindy [difficult]” | Game Design and Player |
|  | Social influence | **Self:**  “I am not influenced by the purchasing habits of others”  “I’m pretty good with not following suite”  “No really, no” [when asked if other players purchasing lootboxes influences the decision to buy lootboxes]  **Others:**  “As much as people probably don’t want to admit it, I think it heavily does”  “If you're playing an Overwatch game and you've got the bog standard skin and your favourite character is Mercy, and you love Mercy, you play her all the time and then you see the enemy team and this person has got this really cool Mercy skin you're like “ I want to be that cool”, so then you'll buy a few good boxes to see if you get that one skin”  “…youtubers that do that mystery box thing and telling their viewers to get their parent’s credit cards, which is the worst thing to say to someone”  “… [lootbox practice] probably teaches gambling characteristics”  “[new players] they see all these people beating them through paying more money, so they spend more money and fall deeper into the hole” | **Player** |
|  | Business model | “Once I've paid £60 for a game, I don't want to spend money on top of it”  “Definitely not a fan of that, if they end up doing it like this it will end up being basically like gambling [on games promoting lootbox purchase]”  “If they are premium price, there's really no excuse to use them [lootboxes] honestly, they [game developers] are making enough money”  “[loot boxes] lead developers to making a cash grab instead of a good game  “I despise the loot box practice, not so much because it milks the players' wallets, but because the whole paradigm has made games shallower”  “I think loot boxes paid for with real money should only be in free to play games/mobile games as a way for earning money for the developers”  “I think loot boxes paid for with real money should only be in free to play games/mobile games as a way for earning money for the developers”  “…if their like free to play games, then maybe [when asked about attitudes towards games using lootboxes]  “Yes, I much prefer a system where loot boxes are only atainable through playing, provided the developer doesn't make it incredibly grindy [difficult]” | Parallels with Gambling, Game design, and Player |
| Attitudes towards content | Accepting talk | “I was alright with Overwatch loot boxes because they are mostly cosmetic. So, you don't have to even come in contact with them at all”  “…at the same time if it is only cosmetic, I am kind of ok with it [a game having lootboxes]”  “I have purchased loot boxes in several games, but only those that provide aesthetic changes”  “Keep them cosmetic only to stop tipping the game in favour of certain players [when asked about the ways lootboxes could be improved]  “It is quite nice to open one and get something that is aesthetically pleasing for your character” | Player |
|  | Negative talk | “…. I really don't like pay to win, it should never feel like you’ve bought half a game, or you’ve bought a game that you have to spend money to do well in it”  “I don't like it to feel pay to win, I want it [lootboxes] to feel like it is an option.”  “I am very adamantly against any kind of loot boxes that makes you stronger in a game, very against that”  “Cosmetic ones are fine but it’s the ones that give you buffs and boosts by letting you spend money in a game is just ridiculous because you put them and they're saying “hey you can get ahead of the competition by just giving us money”  “If you can’t afford them you as a player feel cheated out because other players will have an advantage over you” | Game Design and Player |
|  | Lack real-life value | “…the difference between a loot box in a game and a card pack in real life is that you’re actually getting something physical and tangible. And worst-case scenario you get a card you didn't want but it's rare, you can sell that”  “…the thing is that's a physical thing [buying real life trading card game pack] which you can collect, like the old Pokémon cards still go for a ridiculous amount of money, so there's that aspect of having the cards just to collect… a physical card to me is kind of worth more than a JPEG [digital image]”  “…if you get what you don't want, right, you can literally sell what you don't want on the Steam market and that gives you Steam wallet and CS GO doses as well, you can sell it for Steam wallet and then you can literally use that as real money”  “I like buying them [physical card packs] and receiving physical items because usually they’re just something nice and pretty, and I always have a chance of getting something nice, unlike lootboxes.”  “I'm more likely to buy card booster packs or physical blind boxes as I'm already a collector of similar physical items, rather than a digital item I may never use” | Player |
|  | Cosmetic items | “I was alright with Overwatch loot boxes because they are mostly cosmetic. So, you don't have to even come in contact with them at all”  “…at the same time if it is only cosmetic, I am kind of ok with it [a game having lootboxes]”  “If it's not gambling for money its gambling for digital stuff. It's the same thing”  “I have purchased loot boxes in several games, but only those that provide aesthetic changes”  “Keep them cosmetic only to stop tipping the game in favour of certain players [when asked about the ways lootboxes could be improved]  “It is quite nice to open one and get something that is aesthetically pleasing for your character”  “Cosmetic ones are fine but it’s the ones that give you buffs and boosts by letting you spend money in a game is just ridiculous because you put them and they're saying “hey you can get ahead of the competition by just giving us money” | Player |
